# A computational investigation into rate-dependant vectorcardiogram changes due to specific fibrosis patterns in non-ischæmic dilated cardiomyopathy

**DOI:** 10.1016/j.compbiomed.2020.103895

**Published:** 2020-08

**Authors:** Philip M. Gemmell, Karli Gillette, Gabriel Balaban, Ronak Rajani, Edward J. Vigmond, Gernot Plank, Martin J. Bishop

**Affiliations:** a King’s College London, St. Thomas’ Hospital North Wing, London, SE1 7EH, UK; b Medical University of Graz, Division of Biophysics, Neue Stiftingtalstraße 6(MC1.D.)/IV, 8010 Graz, Austria; c University of Oslo, Research Group for Biomedical Infomatics, Gaustadalléen 23B 0373 Oslo, Norway; d University of Bordeaux, IHU Liryc, Site Hopital Xavier Arnozan, Avenue de Haut-Leveque, 33604 Pessac, France

**Keywords:** Vectorcardiogram, Non-ischæmic dilated cardiomyopathy, Scar, Fibrosis, Conduction slowing, Computer modelling, Random forests

## Abstract

Patients with scar-associated fibrotic tissue remodelling are at greater risk of ventricular arrhythmic events, but current methods to detect the presence of such remodelling require invasive procedures. We present here a potential method to detect the presence, location and dimensions of scar using pacing-dependent changes in the vectorcardiogram (VCG). Using a clinically-derived whole-torso computational model, simulations were conducted at both slow and rapid pacing for a variety of scar patterns within the myocardium, with various VCG-derived metrics being calculated, with changes in these metrics being assessed for their ability to discern the presence and size of scar. Our results indicate that differences in the dipole angle at the end of the QRS complex and differences in the QRS area and duration may be used to predict scar properties. Using machine learning techniques, we were also able to predict the location of the scar to high accuracy, using only these VCG-derived rate-dependent changes as input. Such a non-invasive predictive tool for the presence of scar represents a potentially useful clinical tool for identifying patients at arrhythmic risk.

## Highlights

• Non-invasive assessment of scar arrhythmic risk remains a significant challenge.
• Rate-dependent changes in vectorcardiograms convey the functional presence of scar.
• Random forest machine-learning allows high accuracy for determining scar location.

## Graphical abstract

**Figure:**
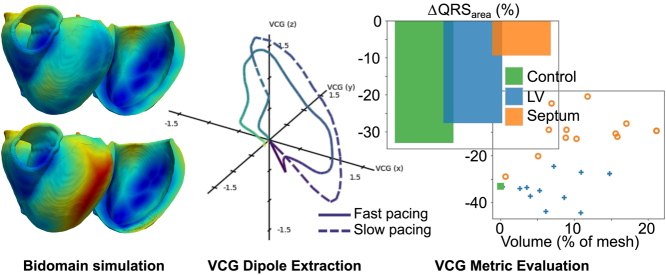

## 1 Introduction

Non-ischæmic cardiomyopathy (NICM) is often associated with scar visible on late gadolinium enhanced (LGE) cardiac magnetic resonance (CMR) imaging. These regions of (micro)-structural fibrotic tissue remodelling are thought to underlie the increased risk of ventricular arrhythmic events and associated sudden cardiac death in this population. Nonetheless, non-invasively identifying these pathological structural changes, and importantly associating them with pathological electrophysiological function in order to stratify risk for implanted device in NICM remains a significant challenge [1].

Regions of fibrosis in NICM are distinct from patterns in ischæmic diseases, being most often patchy or diffuse, and rarely compact [2]. Consequently, they generally do not present a complete barrier to electrical activation, and wavefronts can propagate through these areas, albeit being delayed and disrupted [3]. A key factor determining the arrhythmogenic risk posed by a particular scar substrate appears to be, not necessarily such an activation delay, but more so the enhanced rate-dependent activation delay. In our previous computational modelling study, we demonstrated that a sudden increase in transeptal activation time (directly across a region of midwall fibrosis, as measured clinically [3]) between steady-state and at fast pacing was correlated to the ability of that scar to sustain an induced re-entrant circuit [4]. Mechanistically, modelling uncovered that the increased stress of rapid pacing left tissue relatively refractory, augmenting conduction slowing and isolated regions of unidirectional block as wavefronts traverse the tortuous conduction pathways through these complex patchy fibrotic regions.

The importance of rate-dependent conduction slowing through scar in NICM was further underscored by a recent clinical study by the Leiden group [5]. Here, they reported that the increase in QRS duration (QRSd, used as a measure of ventricular depolarisation) between pacing at baseline and at the effective refractory period under programmed electrical stimulation (PES) could be used to identify those NICM patients in which ventricular tachycardia (VT) could be reliably induced. However, PES is a highly invasive strategy which may be unsuitable for many patients; ideally, a non-invasive ECG-based biomarker would provide a faster and widely-applicable strategy for assessing initial risk of arrhythmia in this population.

**Fig. 1:**
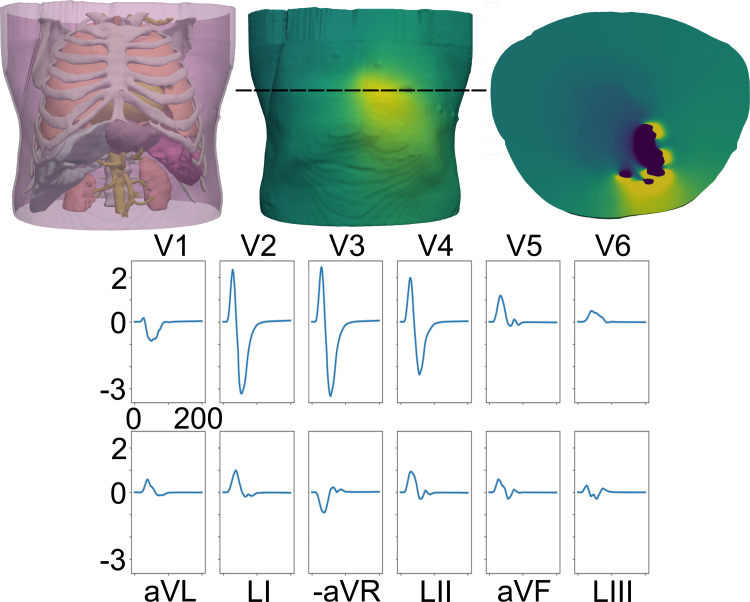
The computational mesh used in this study. *Top Left:* Image of the whole torso mesh, with transparency added to enable viewing of the bones, lungs, kidneys, liver, spleen, stomach and major arteries. *Top Middle:* Example of the electrical potential on the surface of the skin at a point during ventricular depolarisation/repolarisation. *Top Right:* Slice through the torso at the level indicated by the dashed line on the middle image at the same time point as the centre image, showing the electrical potential distribution throughout the torso, including the heart. *Bottom:* ECG trace for the case of no scar, paced at a basic cycle length of 600 ms. Since only ventricular activation is simulated, only the QRS complex of the ECG is reproduced.

Recently, the vectorcardiogram (VCG) has shown something of a resurgence. The VCG, which can be obtained from the standard 12-lead ECG by well characterised transformations [6], provides a means of measuring the total (mean) electric dipole (as a vector) throughout the cardiac cycle. A series of recent works have shown that the beat-to-beat variability in the average VCG dipole during repolarisation is strongly correlated with risk of ventricular arrhythmias in a range of different cardiomyopathies [7], [8]. VCG derived metrics have also been used to identify the presence of myocardial infarction scar [9], [10].

Despite this pioneering work on the use of the VCG to understand repolarisation heterogeneity, its use for quantifying differences in electrical activation sequences has yet to be realised. We therefore hypothesised that quantitative VCG analysis during cardiac depolarisation might be used to assess rate-dependent differences in electrical activation sequences, driven by patchy fibrotic regions, providing the potential for non-invasive risk assessment of arrhythmogenic fibrosis patterns in NICM. To investigate this hypothesis, we used detailed computational whole torso-cardiac models to simulate the effects of different NICM fibrosis patterns on the VCG during sinus activation at different physiological rates. Rate-dependent changes in derived VCG metrics were related to different fibrosis burdens and electrical properties.

Finally, we investigated the potential of machine-learning to make greater use of the available data. Computational modelling presents a uniquely useful tool in this regard, permitting comprehensive testing of a variety of potential VCG metrics in a simulated environment and evaluated using machine-learning techniques, before they are subsequently tested in more focused manner in a clinical environment.

## 2 Materials & methods

### 2.1 Model construction & simulation

Simulations were conducted using a mesh derived from high resolution whole-torso computed tomography (CT) imaging data, previously used by our group [11]—for full details of the mesh generation and parameterisation of the model, see that work. Briefly, CT data were segmented into major organs of interest (skin, skeletal muscles, fat, bones, lungs, spleen, liver, stomach, kidney, major blood vessels) using Seg3D software ( www.sci.utah.edu/software/seg3d.html), with higher resolution contrast CT images being used to derive a more detailed representation of the heart using the Siemens Axseg v4.11 automated segmentation tool [12]. These two segmentations were then combined, and converted to a tetrahedral mesh using the Tarantula meshing software [13]. The resulting mesh is illustrated in the top left panel of Fig. 1. Fibres were introduced to the myocardium using a rule-based method [14], while other tissues were assumed to be homogeneous resistors with negligible capacitance, with respective conductivities given in [11].

### 2.2 Scar generation

**Fig. 2:**
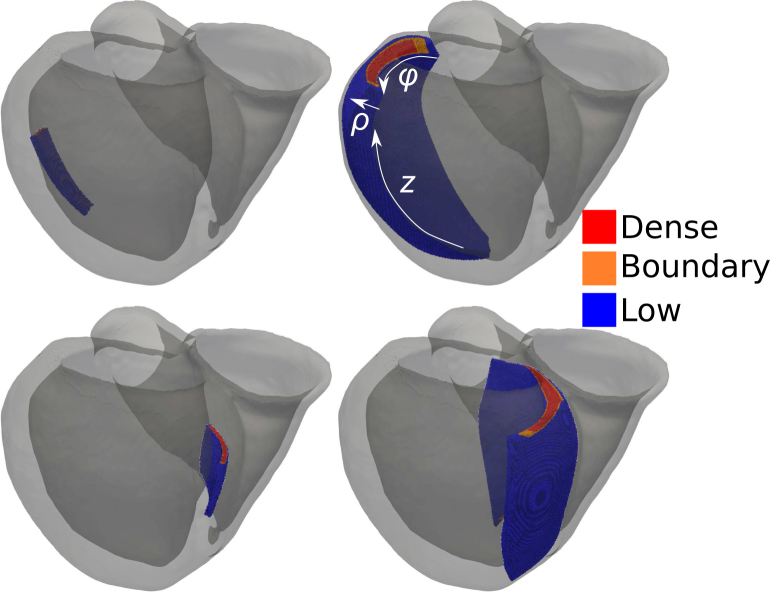
Sizes of small *(left)* and large *(right)* scars in the LV free wall *(top)* and septum *(bottom)*. The UVC parameters used to define scar sizes (ϕ: rotational, ρ: transmural and z: apicobasal) are illustrated on the top right image, corresponding to the largest LV scar, with arrows indicating the direction of parameter increase. ϕ ranges from 0 in the middle of the septum to π∕2 at the outer surface of the anterior left/right ventricular junction, to π in the LV free wall; the inverse scale occurs in the posterior LV. ρ ranges from 0 on the endocardial surface to 1 on the epicardial surface. z ranges from 0 at the apex to 1 at the base.

Within the generated mesh, it was then required to insert representations of scar that were measurable and reproducible. This was achieved using a universal ventricular co-ordinates (UVC) system that provide unique co-ordinates for any point in a smooth heart mesh [15]. The co-ordinates that were varied to determine the dimensions of the scar were: ϕ, which determines the rotational aspect of the scar round the apicobasal axis of the heart (±π in left ventricular (LV) free wall, 0 in septum); ρ, which determines the transmural extent of the scar (0 on endocardial surface, 1 on epicardial/RV endocardial surface); and z, which determines the extent along the apicobasal axis (0 at apex, 1 at base). These parameters are illustrated in Fig. 2.

Two scar locations were selected to be within the midmyocardial inferlateral LV free wall and the midmyocardial septum, both with a basal tendency, to reflect the most prevalent fibrotic regions found in NICM patients [16], [17], [18], [19], [20]; scar in the LV free wall will be referred to as LV scar, whereas scar in the septum of the LV will be referred to as septal scar. Fig. 2 highlights the largest and smallest scars represented in these two locations. Using the UVC definition, the geometries of these scars could be easily altered using maximum ranges of $\frac{\pi }{2}\leq \phi_{\text{LV}}\leq \pi ,-1\leq \phi_{\text{septum}}\leq 1,0.1\leq \rho \leq 0.9,0.3\leq z\leq 0.9$. The generated scar volumes cover the range observed clinically [19], [21], [22], while also allowing us to explore effects outwith this range.

Within the limits defining the scar region, replacement fibrosis (as common in NICM) was introduced to the myocardial mesh by removing elements from the mesh, reproducing the non-conductivity within scar [4]. The probability of a given mesh element being removed was given according to whether the element was classed as existing in a central core zone (with high scar density, $p_{\text{dense}}$), a boundary zone ($p_{\text{BZ}}$), or a low density outer region of scar ($p_{\text{low}}$). This stratification of scar into three regions correlates with observations regarding the density of scar [4]. These regions are illustrated in Fig. 2, where red corresponds to dense scar (central $\frac{2}{3}$ of the scar), orange to boundary zone (central $\frac{5}{6}$ of the scar) and blue to low density outer region of scar. The probabilities for replacement fibrosis were set according to $p_{\text{dense}}$ =0.9, $p_{\text{BZ}}$ =0.75 and $p_{\text{low}}$ =0.6 (save for simulations where the effect of scar density was being assessed).

### 2.3 Model simulation

Electrophysiological activation was simulated in the mesh with the Cardiac Arrhythmia Research Package (CARP) [23]. The cellular action potential was modelled based on the ten Tusscher model [24], with tissue conductivities from [25] (save for spinal cord [26] and spleen [27]). Heart tissue conductivies were based on those used in [4], which were tuned to reproduce conduction velocities from [28], with slight tuning to reproduce realistic ECG morphologies and QRS durations within physiological ranges during sinus beats. The transverse conductivities were reduced by 50% in scar, and the longitudinal conductivities reduced by 25% and 50% for intermediate and dense scar, respectively, consistent with [28] which reported a correlation between fibrosis level and conduction velocity slowing. The same ionic model is used in scar as in healthy myocardium. As the focus of our study is for ventricular depolarisation, atrial activation was not considered. Approximate sinus activation of the ventricles was achieved through pacing at 56 distinct locations on the LV endocardium with individual site activation times derived from experimental data, to reproduce the activation of the LV via the Purkinje network; these pacing locations were based on [29]. In order to assess the effects of the rate-dependent conduction velocity slowing, simulations were conducted for slow pacing (basic cycle length (BCL) =600ms) and for rapid pacing (BCL =300ms). In both cases, the meshes were ‘pre-conditioned’ by pacing at the given BCL for 5 beats—these were simulated using the monodomain equations, for increased computational tractability. Once this pre-conditioning was complete, the mesh was simulated with one final stimulation for a further 200 ms—this final simulation was conducted using a solution of a pseudo-bidomain formulation, involving infrequent solves of the elliptic problem, permitting extraction of the electrical potential across the torso, and subsequent extraction of the ECG [30]. On a 128 core supercomputing cluster, these simulations took ∼3 h.

### 2.4 Data analysis

After simulation, the electrical potential on the torso surface was extracted at points corresponding to the lead locations in a standard 12-lead ECG setup—an example ECG trace for no scar at a BCL of 600 ms is shown in Fig. 1. The corresponding VCG is then reconstructed from the ECG by means of a linear combination of ECG data; this is done by the Kors matrix multiplication [6] such that the z-component corresponds to the long axis of the body, and the x-component is the anterior–posterior, such that the xy-plane is the transverse plane, and similarly the xz-plane is the frontal plane and the yz-plane is the saggital plane. The start point of the QRS complex was calculated by finding the point at which the spatial velocity of the filtered VCG (where the spatial velocity is defined as the Euclidean norm of the velocity in each of the x, y and z directions) exceeds a threshold value (here set to be 0.15 of the maximum spatial velocity) [31], [32], [33]. The end point of the QRS complex was similarly defined by finding the point at which the spatial velocity exceeded this value, tracking from the end of the VCG rather than the start. All subsequent analysis was conducted on the QRS portion of the VCG.

**Fig. 3:**
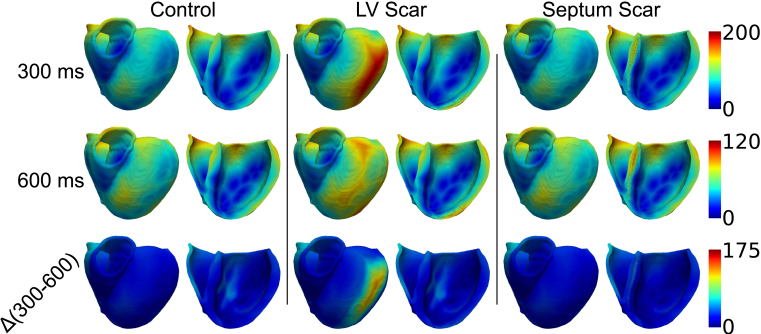
Activation maps for control *(left)*, LV free wall scar *(middle)* and septal scar *(right)*, for BCL of 300 ms *(top)* and 600 ms *(middle)*; the scars are the largest simulated. The difference between the activation maps for 600 ms and 300 ms is shown at the bottom. All activation maps for BCL =300ms, BCL =600ms and for the differences between the two share a colour-scale; these are shown to the right of the rows. In all cases, data are plotted on the epicardial surface, along with clipping planes used to highlight endocardial and midwall data.

#### 2.4.1 Rate-dependent VCG metrics

Several different metrics were calculated, based on either established or potential clinical benefit. Firstly, the QRS complex was determined, with the duration being calculated (QRSd); all other metrics, save those for specific time-points, were based on the QRS complex. The area under the VCG curve during QRS (QRS${}_{\text{area}}$) is calculated as the ${\text{QRS}}_{\text{area}}^{2}={\text{QRS}}_{\text{area},x}^{2}+{\text{QRS}}_{\text{area},y}^{2}+{\text{QRS}}_{\text{area},z}^{2}$, where QRS${}_{\text{area},x}$ is the area between the x-component of the VCG dipole loopVCG loop in the x-direction and the baseline [34], [35], [36], [37]. The weighted average azimuthal (WAA) and weighted average elevation (WAE) angles are the average azimuthal (angle between the vector on the transverse plane and the x-axis) and elevation angles (angle between the vector and y-axis), weighted according to the dipole magnitude [7]. The mean dipole magnitude ($\bar{\text{VCG}}$) and the maximum dipole magnitude (VCG${}_{\text{max}}$) were also recorded, along with the time at which the maximum dipole was measured ($t_{\text{VCGmax}}$). These metrics were all specifically computed at fast and slow pacing, and the difference between the two examined—these differences are annotated using a Δ, *e.g.* ΔVCG${}_{\text{max}}$, and their values are expressed as percentage differences compared to their values at slow pacing. Finally, the angle difference between fast and slow pacing for the mean weighted dipoles ($dT_{\text{VCGmean}}$), the maximum magnitude dipoles ($dT_{\text{VCGmax}}$) and the dipoles at QRS start, mid-point and end ($dT_{\text{QRSstart}}$, $dT_{\text{QRSmid}}$ and $dT_{\text{QRSend}}$, respectively) were recorded. These angular metrics are expressed in degrees.

#### 2.4.2 Classification using random forests

Random forests [38], [39], [40], [41] were used to determine whether a combination of measured metrics can be used to meaningfully predict properties of the scar. Random forest analysis represents an attractive machine learning technique in this instance, taking advantage of the easily understandable heuristic of decision trees, while minimising the risk of individual decisions trees to over-fit to the data. The ‘bagging’ of data also permits an efficient use of the limited data available in training the model. Of further use in an investigation such as ours, where several metrics are being assessed for their utility, random forests permit identification of which of these variables are of greater import for classification, based on their overall import in the forest.

The random forests analysis was implemented using the scikit-learn Python package [42], using a classifier to determine whether the scar was present in the LV free wall or not, or in the septum or not (these were independent, binary questions); analysis was conducted with a random seed value of 42 to ensure reproducibility. In total, 30 simulations with unique scar parameters were available to train the foests. The individual trees in the forest were created to maximise the homogeneity of the population after the question, as measured by the Gini coefficient. Random forests were trained and evaluated using both 5-fold and n-fold cross-validation, for random forests of both 20 and 1000 trees; no limit was based on the number of nodes in the trees. For the former, this involved splitting the data into five segments, then performing five rounds of training/testing, with each segment being the testing set for one of the rounds. For the latter, one datum is used for testing over n rounds of training/testing, with n being the number of data. All metrics available for selection as node choices in the decision trees in the random forest, with the relative importance of the metrics in these forests being judged using the methods of the scikit-learn package. Random forest regression was also attempted for the scar parameters, but these results were less promising, and less readily translatable to geometrically complex real-world scar geometries than the simpler classification problem.

## 3 Results

Initially, we examine the effect of scar upon ventricular activation and the corresponding effect that this has on the VCG and important derived metrics; those metrics judged to not be important are discussed in the Supplementary Information. Here, a large (default) sized scar is represented to maximise the effects, and results compared to the control (scar free) case.

We then examine the sensitivity of the VCG and derived metrics when the size and shape of the scarred regions is systematically altered. To assess the effect of the shape of scar, we used 4 ranges of ϕ for LV scar and 5 ranges of ϕ for the septal scar, and 5 ranges of ρ and 4 ranges for z for both LV and septal scar. The effect of the size of the scar was assessed by using all resulting simulations, and calculating the resulting scar volume/surface area. The density of fibrosis was assessed using 3 different density ranges for dense, intermediate and border zone scar densities.

Finally, we examine the utility of random forests to determine the location of scar from the measured VCG metrics.

### 3.1 Pacing rate dependence of scar on ventricular activation maps

Fig. 3 shows activation maps derived during fast (BCL = 300ms, top row) and slow (BCL = 600ms, middle row) pacing, along with the difference between the two (bottom row) for control (left), LV scar (middle) and septal scar (right); the scars are the largest simulated.

The maximum activation time when no scar is present (158.7ms at 300ms BCL, 102.9ms at 600ms BCL) is almost always less than when scar is present in either the LV free wall (>200ms at 300ms BCL, 102.9ms at 600ms BCL) or the septum (172.2ms at 300ms BCL, 115.4ms at 600ms BCL). This is similarly true for the difference between activation times for fast and slow pacing: the difference between fast and slow pacing activation times is up to 65.6ms for control, whereas this increases to 96.3ms for septal scar and 174.4ms for LV scar (there is not a direct correlation between the point of final activation at fast and slow pacing, hence the activation time difference is not a direct subtraction).

**Fig. 4:**
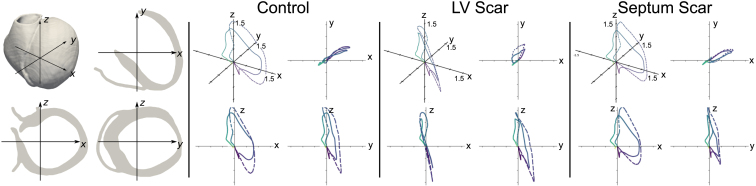
Orientation of mesh with respect to x, y and z axes *far left*. VCG traces for control *(second from left)*, LV free wall scar *(second from right)* and septal scar *(far right)*; the traces for fast pacing (BCL = 300 ms) is represented by a solid line, the trace for slow pacing (BCL = 600 ms) by a dashed line. In each group, the 3D representation of the VCG is shown in the top left corner, with the x-y plot shown top right, the x-z plot bottom left, and the y-z plot bottom right; all axes range −1.5→1.5.

Visually, these changes can be subtle to observe, save for the noticeable delay in activation of the LV free wall in the presence of LV scar, which is more pronounced at faster pacing rates. At slower pacing rates, the slowed activation pattern has a more complex relation to scar location, as the activation now proceeds both around and through the scar (while at fast pacing rates, the activation delay is solely due to the activation wave progressing through the scar). The differences between the septal scar and the control, however, are ‘hidden’, with the delayed activation differences at both fast and slow pacing being in the mid-septum, which would be inaccessible to measurement in a clinical setting.

The differences between the activation maps for fast and slow pacing can be similarly subtle, but differences between control and scar cases can be brought out. The difference map between fast and slow pacing is relatively minor, with no scar to influence the activation pattern during either fast or slow pacing. The epicardial delay in the LV due to LV scar remains evident. The changes in the activation pattern due to the presence of septal scar is difficult to notice visually, as the major changes occur in the septum, with resulting changes in the activation time of the right ventricle.

### 3.2 Pacing rate dependent effect of scar on VCG loops

Due to the subtlety of the changes in activation maps, changes due to the presence or absence of scar are more easily tracked and determined by changes in the VCG, which can show more marked changes—VCG plots are shown in Fig. 4. The very nature of the VCG is to track the motion of the electrical dipole that results from the distribution of electrical charge over the myocardium. Changes in the depolarisation sequence (including those caused by scar) will necessarily impact on the dipole movement, and thus will affect the VCG.

There is relatively little change in the VCG loop between slow and fast pacing when no scar is present, save for an exaggeration of the loop in the y and z directions. In the presence of scar in the LV free wall, the progression of the VCG in the positive x-direction is severely curtailed—this is evident at both fast and slow pacing. The trajectory of the VCG dipole is also more complicated, with a ‘loop’ evident in the xz plane. The presence of scar in the septum curtails the trajectory of the dipole in the negative z direction at slow pacing rates compared to control.

### 3.3 Quantifiable rate-dependent changes in VCG-metrics influenced by scar

**Fig. 5:**
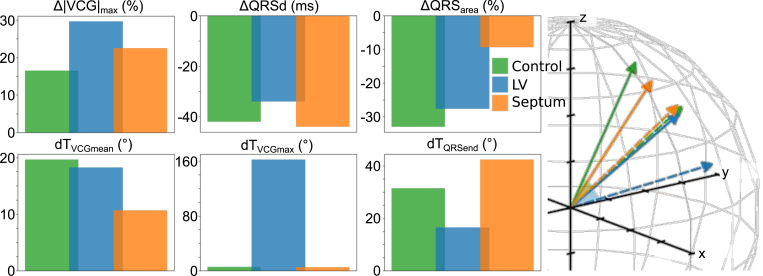
Changes in metrics between fast and slow pacing *(left)*. Changes in the maximum dipole and QRS area are expressed as percentage changes from their value for BCL =600ms. The mean unit weighted dipoles for the VCG during QRS are shown *(right)*; a solid line represents the value for BCL =300ms, a dashed line for BCL =600ms.

In quantifying the effect of scar on the VCG, the difference exhibited in six metrics between slow and fast pacing were established to demonstrate a non-negligible reaction to the presence of scar: these metrics are maximum magnitude of the VCG dipole (ΔVCG${}_{\text{max}}$), QRS area (ΔQRS${}_{\text{area}}$), QRS duration (ΔQRSd) and angular difference between fast and slow pacing for mean weighted dipoles ($dT_{\text{VCGmean}}$), maximum dipoles ($dT_{\text{VCGmax}}$) and dipoles at the end of QRS ($dT_{\text{QRSend}}$). The effect of the presence of the largest simulated scar (for both LV free wall and septum) on the differences for these metrics is shown in Fig. 5, along with an illustration for the mean weighted dipoles at fast (solid arrow) and slow (dashed arrow) pacing for control, LV and septal scar conditions, to demonstrate the changes in $dT_{\text{VCGmean}}$.

Under control conditions, there is very little change observed in VCG${}_{\text{max}}$, resulting in a relatively small delta (16.5%). However, in the presence of scar, this increases (29.6% in LV and 22.5% in septum, when considering the largest scar in both). The change in QRSd is also noticeable for LV scar, though not necessarily for scar in the septum: ΔQRSd for control is −42ms, which reduces to −34ms for scar in the LV free wall (ΔQRSd for septal scar is −44ms). Septal scar has a far more profound effect, on the other hand, for changes in QRS${}_{\text{area}}$: under control conditions, there is a fractional decline in QRS${}_{\text{area}}$ of −33.0% (and a similar decline of −27.6% for LV scar), while this decline is markedly reduced for septal scar to only −9.4%.

$dT_{\text{VCGmean}}$ tends to show a decline in the presence of septal scar, but with mostly marginal differences for scar in the LV free wall. Under control conditions, $dT_{\text{VCGmean}}$ is 19.6°, with a negligible change to 18.3° for the largest LV scar simulated. However, for septal scar, $dT_{\text{VCGmean}}$ decreases further to 10.7°. The angular differences for maximum dipoles for maximum LV scar is more promising for scar detection: for control, $dT_{\text{VCGmax}}$ is 5.8° (with septal scar being 4.9°), compared to a substantial increase to 162.1° for LV scar. The final metric of note, $dT_{\text{QRSend}}$, indicates opposing reaction to scar when the scar is in the septum or LV free wall: the control case shows an angular difference of 31.4°, which decreases to 16.6° for LV scar and increases to 42.4° for septal scar.

### 3.4 Effect of scar size and morphology on rate-dependent changes in VCG metrics

**Fig. 6:**
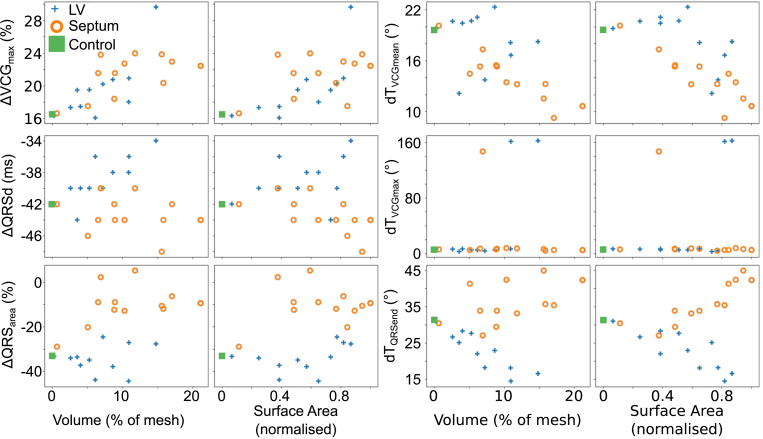
Changes in metrics between fast and slow pacing versus volume and surface area of scar, for both septal and LV free wall scars. ΔVCG${}_{\text{max}}$ and ΔQRS${}_{\text{area}}$ are expressed as percentage changes from slow pacing, ΔQRSd is expressed as the difference between slow and fast pacing, and $dT_{\text{VCGmean}}$, $dT_{\text{VCGmax}}$ and $dT_{\text{QRSend}}$ are expressed as angular differences between the dipole vectors.

**Fig. 7:**
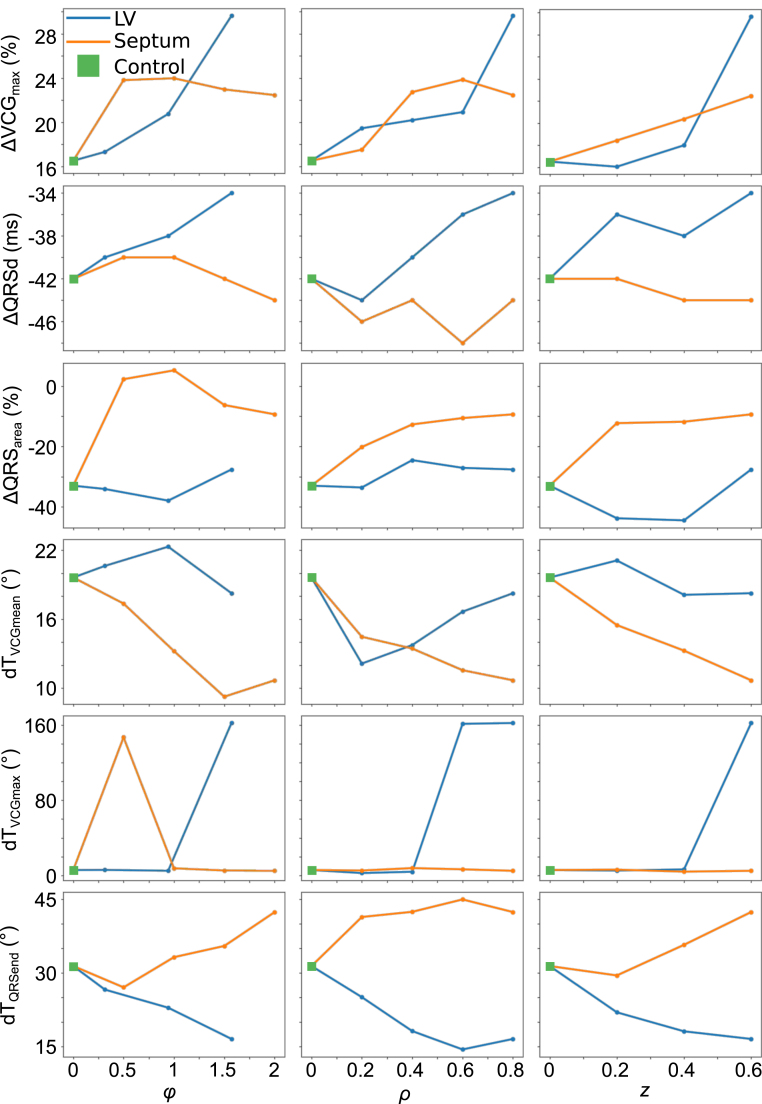
Changes in metrics between fast and slow pacing versus ϕ, ρ and z, for both septal and LV free wall scars. ΔVCG${}_{\text{max}}$ and ΔQRS${}_{\text{area}}$ are expressed as percentage changes from slow pacing, ΔQRSd is expressed as the difference between slow and fast pacing, and $dT_{\text{VCGmean}}$, $dT_{\text{VCGmax}}$ and $dT_{\text{QRSend}}$ are expressed as angular differences between the dipole vectors.

More in depth analysis of the metrics is conducted with more tailored simulations to investigate the individual UVC parameter effects: the effect of scar volume and scar surface area is shown in Fig. 6, whereas the effect of the various scar size parameters (ϕ,ρ,z) is shown in Fig. 7; in the latter, all parameters save the one being considered are set to their maximum value. The effects on each metric shall now be discussed in turn.

#### 3.4.1 Effects on VCG${}_{\text{max}}$

When scar is present in either the septal or free wall of the LV there is almost always an increase in ΔVCG${}_{\text{max}}$, with a stronger degree of correlation with the scar volume rather than the scar surface area (which corresponds to the fact that increased scar volume leads to reduced excitable tissue mass to change VCG${}_{\text{max}}$); this correlation is marginally more apparent for septal scar than LV free wall scar.

For scar in the LV free wall, no particular metric of scar size holds a privileged position in its effect on ΔVCG${}_{\text{max}}$—as the scar is increased in size for any of ϕ,ρ or z, the change from control increases, with this change becoming more marked with larger scars. For scar in the septal wall, the increase in ΔVCG${}_{\text{max}}$ seems to be relatively independent of ϕ (rotational), with the increase in difference scaling instead with ρ (transmural) and z (apicobasal).

#### 3.4.2 Effects on QRSd

ΔQRSd is affected in opposing manners according to whether the scar is in the LV free wall or in the septum. The more noticeable of these changes is for scar in the LV free wall, which causes an increase in ΔQRSd, which is more apparent with scar volume than with scar surface area (Fig. 6, third row). This increase is relatively independent of which UVC parameter changes for scar size, as an increase in any results in an increase in ΔQRSd (save for a transient decrease for small values of z) (Fig. 7).

Septal scar, on the other hand, tends to cause a reduction in ΔQRSd compared to control, but this reduction is less noticeable and less consistent.

#### 3.4.3 Effects on QRS${}_{\text{area}}$

There is a noticeable decrease in ΔQRS${}_{\text{area}}$ under control conditions, with this decrease being relatively unaffected by scar in the LV free wall. However, this decrease is reduced by septal scar (Fig. 6, fourth row). Once again, this increase from control is evident for all UVC metrics of septal scar size (Fig. 7, fourth row). However, while it is almost always true that increasing scar size in each of the UVC parameters leads to an increase in effect, it is not always the case—for instance, changes in ϕ in the septum beyond the range −0.5→0.5 result in a marginal decrease in ΔQRS${}_{\text{area}}$ though the change in QRS${}_{\text{area}}$ remains less than in a scar-free instance.

#### 3.4.4 Effects on angular difference in mean weighted dipole

The angular difference between the mean dipole at fast pacing (BCL = 300ms) and slow pacing (BCL = 600ms) tends to reduce in the presence of scar in the septum, while demonstrating negligible and inconsistent changes for scar in the LV free wall (Fig. 6, top right, Fig. 7, fourth row). For septal scar, the decrease in $dT_{\text{VCGmean}}$ correlates well with both scar volume and scar surface area. The decrease in $dT_{\text{VCGmean}}$ is also relatively linear for all UVC coordinates, with an increase in any of them correlating with a decrease in $dT_{\text{VCGmean}}$ (save for large values of ϕ, but the increase in $dT_{\text{VCGmean}}$ from −0.75≥ϕ≥0.75 to −1≥ϕ≥1 is negligible: 9.3° to 10.7°).

Scar in the LV free wall tends to have a negligible effect on $dT_{\text{VCGmean}}$, ranging between 18.2° and 22.3° for all values of ϕ, and between 18.1° and 21.1° for changes in z. For both parameters, the minimum and maximum ranges for $dT_{\text{VCGmean}}$ do not correlate with the minimum and maximum ranges of the parameter. Of note is the variation in $dT_{\text{VCGmean}}$ for changes in ρ for LV free wall scar—the minimum simulated range of ρ for LV scar results in $dT_{\text{VCGmean}}$ decreasing to 12.1°, which subsequent increases in the ρ decreasing the apparent effect.

#### 3.4.5 Effects on angular difference in maximum dipole

Upon closer examination of the changes in $dT_{\text{VCGmax}}$ with scar size, the promising change in $dT_{\text{VCGmax}}$ noted earlier is demonstrated to a non-reliable metric for presence of scar. Under most circumstances, there is negligible change in $dT_{\text{VCGmax}}$ (ranging from 2.5° to 7.8°), and those conditions under which there is a non-negligible change are unpredictable and rare (two conditions for LV free wall scar and only one for septal scar). The reason for this is due to maximum dipole location: under most circumstances, the maximum dipole occurs relatively early in the QRS, and the relative time of this dipole does not change significantly. As such, while the scar alters the trajectory of the loop, the shift in VCG${}_{\text{max}}$ is relatively consistent between both fast and slow pacing, and the resulting $dT_{\text{VCGmax}}$ is consequently relatively consistent, regardless of presence of scar.

The exception to this consistency of $dT_{\text{VCGmax}}$ is when the time at which VCG${}_{\text{max}}$ occurs shifts during the QRS for either fast or slow pacing: when this shift happens, and VCG${}_{\text{max}}$ instead occurs far later in the VCG, the angular difference is thus large. As such, the observed large values of $dT_{\text{VCGmax}}$ can more accurately be considered due to a change in the *timing* of VCG${}_{\text{max}}$, rather than a change in VCG${}_{\text{max}}$ itself.

#### 3.4.6 Effects on angular difference at QRS end

The effect of scar on $dT_{\text{QRSend}}$ is dependent on whether the scar is in the septum or the LV free wall: when in the septum, $dT_{\text{QRSend}}$ tends to increase, whereas when the scar is in the LV free wall, $dT_{\text{QRSend}}$ decreases. This effect is more strongly correlated for LV free wall scar, especially when considering scar volume, while the correlation is less substantial for septal scar. The effects for LV scar are consistent for the scar size parameters: under almost every situation, an increase in scar size parameter corresponds to an increase in the effect of scar on $dT_{\text{QRSend}}$. The same cannot be said of septal scar: small septal scars (when considered via ϕ and z) will result in a marginal decrease in $dT_{\text{QRSend}}$, and it is only when the scar increases in size does the noted increase in $dT_{\text{QRSend}}$ occur. Septal scar looks to be relatively insensitive to changes in ρ, with only marginal changes to $dT_{\text{QRSend}}$ for any change to ρ once scar is present.

### 3.5 Effect of fibrosis density on rate-dependent changes in VCG metrics

**Fig. 8:**
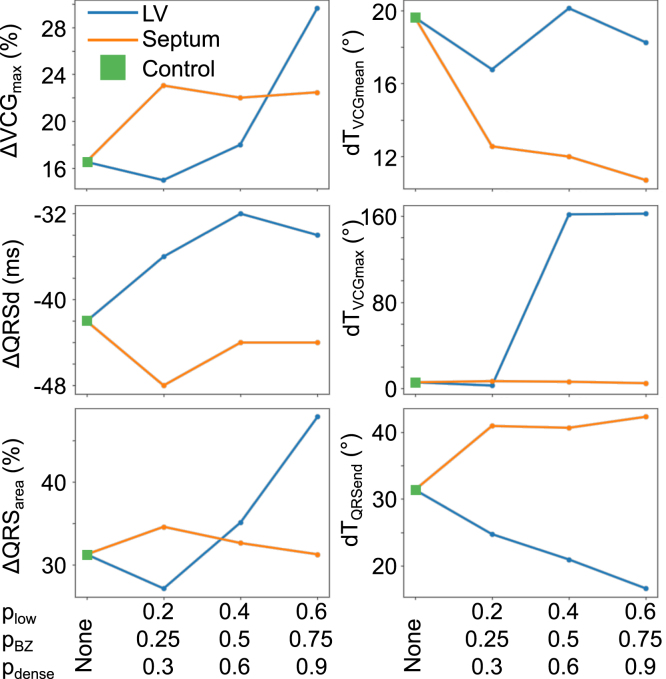
Effect of scar density on the changes in dipole maximum magnitude ΔVCG${}_{\text{max}}$ *(top left)*, ΔQRSd *(middle left)*, ΔQRS${}_{\text{area}}$ *(bottom left)*, $dT_{\text{VCGmean}}$ *(top right)*, $dT_{\text{VCGmax}}$ *(middle right)* and $dT_{\text{QRSend}}$ *(bottom right)*.

The effects of scar density on the noted metrics, shown in Fig. 8, are complicated, and do not always indicate a linear relationship between scar density and scar effects. The effect of septal scar on ΔVCG${}_{\text{max}}$ is relatively unaffected by scar density, but LV free wall scar’s effect is increased by increased density of fibrosis (it can be noted that for very sparse fibrosis, ΔVCG${}_{\text{max}}$ actually decreases for scar in the LV).

Low fibrosis scar again has the greatest effect for septal scar on ΔQRSd, causing the greatest reduction compared to control—subsequent increases in fibrosis density reduce the effect. The effect of density on LV free wall scar is more consistent, with increased density of fibrosis leading to increased changes in ΔQRSd compared to control (save for the most dense fibrosis, though that remains increased compared to control).

The effect of septal scar density on ΔQRS${}_{\text{area}}$ is negligible. The effect of fibrosis density for LV free wall scar on ΔQRS${}_{\text{area}}$ is similar to the effect on ΔVCG${}_{\text{max}}$: an initial reduction in the effect compared to control, followed by consistent increase in effect to maximum effect for maximum density.

The effect on scar density on $dT_{\text{VCGmean}}$ is not consistent for LV scar, with no consistent relation between scar density and effect on $dT_{\text{VCGmean}}$—the greatest effect on $dT_{\text{VCGmean}}$ is for sparse fibrosis for LV free wall scar, with intermediate scar density causing an increase in $dT_{\text{VCGmean}}$ (the opposite trend for all other observations of $dT_{\text{VCGmean}}$ with LV free wall scar). On the other hand, scar density has a consistent effect on $dT_{\text{VCGmean}}$ for septal scar, with increasing density resulting in an increasing effect (greater decrease in $dT_{\text{VCGmean}}$).

For reasons previously mentioned, scar density has a negligible effect for septal scar effects on $dT_{\text{VCGmax}}$, with there being a minimum threshold of scar density for the ‘shift’ in VCG${}_{\text{max}}$to become apparent for LV scar; note, however, that for the same reasons that scar size is not necessarily easily linked to the shift in VCG${}_{\text{max}}$, it is difficult to link the effect to scar density.

The changes in $dT_{\text{QRSend}}$ for LV free wall scar are relatively linear, with increasing density resulting in increasing decline of $dT_{\text{QRSend}}$. For septal scar, however, the effect of density is not so clear, with the increase in $dT_{\text{QRSend}}$ being relatively insensitive to increasing density, with only marginal increase in effect for increase in scar density.

### 3.6 Scar localisation

It was then assessed how accurately the location of a scar could be determined—given that a scar is present (*i.e.* the control case is excluded from this part of analysis), can the rate-dependent change in the metrics be used to determine whether the scar is in the LV free wall or the septum? This was done using receiver operating characteristic (ROC) curve to determine the sensitivity/specificity of the metric in discriminating between LV and septal scars, with the area under curve (AUC) used to judge the relative merit of the metrics; the results are shown in Table 1. Each metric was incrementally increased from its minimum to its maximum value, and each observation was labelled as either true or false positive or negative, depending on whether the measurement correlated to a scar in the LV free wall or not. The resulting true positive rates and false positive rates then provided the assessed ROC curves.

The AUC analysis indicates that, while the QRS metrics may not necessarily provide information regarding the scar dimensions, almost all of them provide some level of indication of scar location. The rate dependent changes in maximum VCG, measured in terms of both magnitude and angular difference, are relatively insensitive to discrimination between LV and septal scars, performing only marginally better than random chance. $dT_{\text{QRSstart}}$ is similarly non-discriminatory. The remaining metrics, on the other hand, provide relatively accurate means of discriminating scar location. This is especially true for $dT_{\text{QRSend}}$: this can be seen in Fig. 6, wherein the diverging effect of scar in LV and septum provides for a very effective threshold to discriminate between LV and septal scars.

**Table 1:** Area Under Curve (AUC) for the receiver operator characteristics (ROC) curves when using the specified metric to discriminate between scar in the LV and the septum. These are given for metrics identified to show correlation with scar size *(left)* and those that do not show correlation *(right)*.

Metric	AUC	Metric	AUC
ΔVCG${}_{\text{max}}$	0.61	$\bar{\text{VCG}}$	0.87
ΔQRSd	0.08	WAA	0.88
ΔQRS${}_{\text{area}}$	0.97	WAE	0.89
$dT_{\text{VCGmean}}$	0.11	$t_{\text{VCGmax}}$	0.70
$dT_{\text{VCGmax}}$	0.40	$dT_{\text{QRSstart}}$	0.38
$dT_{\text{QRSend}}$	0.98	$dT_{\text{QRSmid}}$	0.75

Table 2 shows the accuracy of the random forest in predicting whether the scar is in the LV or the septum, depending on whether the random forest is composed of either 20 or 1000 trees, and whether the cross-validation is 5-fold or n-fold; there was little change in results if the control case was excluded from training/testing. By assessing random forests of sizes between 5 and 60, it was established that the out-of-bag error does not change substantially for classification for forests larger than ∼20. Nevertheless, the larger forests tended to produce more accurate results when subject to cross-validation.

**Table 2:** The accuracy of a random forest, as measured using 5-fold and n-fold cross-validation for random forests of 20 and 1000 trees.

Output	Accuracy (mean ± std)			
	20 trees		1000 trees	
	5-fold	n-fold	5-fold	n-fold
LV	76.67±13.33%	90.71±15.22%	83.33±14.91%	90.71±15.22%
Septum	83.33±10.54%	90.00±11.65%	86.67±12.47%	93.57±10.25%

**Fig. 9:**
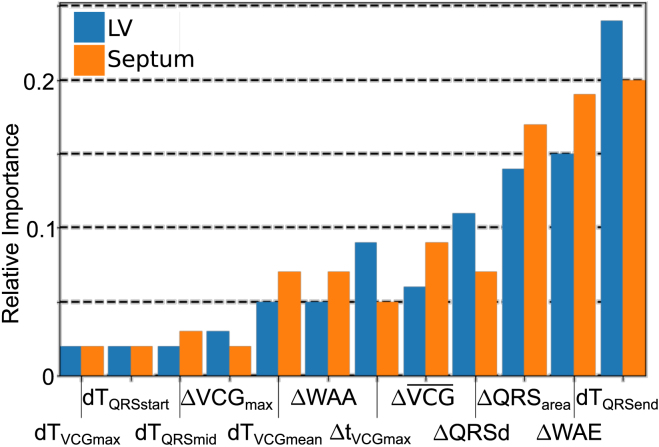
Relative importance of each of the measured metrics in the random forests to classify scar location as either LV or septum.

Fig. 9 shows the relative importance of each measured metric to the random forests. There is a relatively high degree of correlation between importance for judging whether the scar is in the LV, or whether the scar is in the septum. The most important metrics for random forests are $dT_{\text{QRSend}}$ and ΔWAE, which individually demonstrated no particular ability to distinguish scar. Conversely, it can be noted that ΔVCG${}_{\text{max}}$ (one of the prominent individual markers for scar presence, regardless of LV/septal location) is relatively unimportant for random forest decisions. These results indicate which of several clinically measurable metrics are potentially useful, and which can likely be disregarded before clinical evaluation.

## 4 Discussion

Clinical tools available to non-invasively assess arrhythmic risk in NICM patients are currently inadequate, with resulting difficulties to determine best clinical practice [1]. In this work, we investigate the potential of rate-dependent VCG-derived metrics to identify arrhythmogenic conduction slowing through regions of fibrotic remodelling in the NICM heart. Our study highlights the utility of rate-dependent changes in the QRS end dipole angle ($dT_{\text{QRSend}}$) as the VCG metric most able to identify the significant changes in activation pattern, and furthermore differentiate between scar location in either the septum or LV free wall. Further potential utility for rate-dependent changes in the maximum dipole magnitude (VCG${}_{\text{max}}$), QRS duration (QRSd), the area under the VCG curve during the QRS complex (QRS${}_{\text{area}}$), and the dipole angle difference between mean QRS dipole was established ($dT_{\text{VCGmean}}$), with potentially useful, if not reliably consistent changes in the dipole angle between the maximum QRS dipole also noted ($dT_{\text{VCGmax}}$). The application of machine learning techniques (in this instance, random forests) indicate a promising avenue for clinical identification of scar location based on electrophysiological changes, using a combination of metrics that individually would not be conclusive. Of equal utility for future clinical investigation are the results indicating the lack of utility of other metrics. These results for such a clinically relevant outcome indicate that further investigation is warranted, expanding to more realistic scar geometries.

### 4.1 Identifying fibrotic conduction slowing in NICM

LGE CMR has demonstrated significant success at identifying myocardial fibrosis within NICM patients, and, more importantly, deriving quantitative LGE-derived metrics which are strongly associated with arrhythmic events and sudden cardiac death in these patients [16]. One of the key benefits of LGE CMR is that it represents a non-invasive technique to assess the scar burden of a patient. However, it identifies regions of structural fibrotic remodelling, without necessarily informing how this remodelling affects the (patho-)electrophysiological function of the myocardium. A method to directly measure the electrophysiological changes caused by scar and fibrotic remodelling, while maintaining the non-invasive benefits of LGE CMR, thus represents a useful clinical tool to complement existing imaging modalities.

Our recent detailed computational modelling investigations have suggested that the initiation of re-entrant activity in NICM patients may be due to significant conduction slowing and isolated regions of unidirectional block, localised within the scarred regions [4], which is sensitively dependent upon the specific density [43] and shape [44] of the scar substrate. Significant conduction delays, quantified by abrupt QRS prolongation under programmed electrical stimulation, have also been shown in a recent clinical study to be strongly associated with VT inducibility [5]. Meanwhile, invasive catheter measurements of the degree of transmural conduction delay (>40ms) through midwall (septal) scar in NICM patients have been associated with the VT substrate [3].

Our own recent modelling work has built on this idea to suggest that the difference in transmural conduction delays between fast and slow pacing is a more robust marker of arrhythmia inducibility, depending as it does on the density of fibrosis which governs the likelihood of conduction block in the scar substrate. Directly accessing (via catherisation) regions of myocardium containing scar to perform invasive pacing and mapping measurements represent non-optimal risk stratification strategies. The non-invasive assessment of rate-dependent conduction slowing via VCG analysis presented here does not have such limitations. Moreover, the potential ability of the VCG dipole analysis (which inherently contains anatomical information regarding direction) to be augmented by LGE information from CMR to focus analysis on specific (known) ventricular scarred regions, represents an additional benefit from general multi-lead QRS-prolongation analysis [5].

### 4.2 Utility of VCG-derived metrics

The VCG is regaining popularity as a means of comprehensively evaluating the electrical activation and repolarisation sequences during a cardiac cycle through visualising, and quantifying, the movement of the net electrical cardiac dipole [45], [46]. VCG analysis has recently been used to facilitate patient-specific personalisation of electrophysiological computational models [47], and VCG-QRS analysis used to assess differences in activation sequences in responders of cardiac resynchronisation therapy [34]. A comprehensive series of recent work has demonstrated the importance of beat-to-beat differences in repolarisation sequences, directly quantified as angular differences in the mean weighted VCG dipole during the T-wave between successive beats ($dT_{\text{VCGmean}}$), which can be used to quantitatively identify patients at arrhythmic risk in a mixed cardiomyopathy patient cohort [7], [8], [34], [48], [49].

Direct T-wave analysis of individual standard ECG leads can often be problematic, due to the relatively low amplitude and the subtle variations in T-wave morphology that are under investigation, which are thought to underlie important arrhythmogenic substrates. Thus, the use of the VCG in this context is a potentially powerful tool as no information is thrown away, maintaining all relevant electrophysiological information regarding the cardiac electrical sequences. The VCG distils down a multitude of complex information in individual ECG-traces into a single metric (the VCG net dipole), with a relatively straightforward interpretation. Here, we have furthered the use of inter-beat differences in the net VCG dipole, but importantly focussing on the net QRS dipole (as opposed to its previous use in the net T-wave dipole [7], [8], [48]), as well as examining differences between fast and slow (sinus) beats. Our analysis has demonstrated the potential utility of the angular differences between the dipoles at fast and slow pacing applied in this context, with the specific aim of extracting the subtle effects of localised rate-dependant conduction slowing, known to be a marker of arrhythmic risk in these patients. These angular differences are most readily and consistently observed as changes in the angular difference at the end of the QRS complex ($dT_{\text{QRSend}}$), but with potentially useful changes also observed in angular differences for the mean dipole ($dT_{\text{VCGmean}}$) and the maximum observed dipole ($dT_{\text{VCGmax}}$). Our work has also indicated the potential benefit of ΔVCG${}_{\text{max}}$, ΔQRSd and ΔQRS${}_{\text{area}}$ as potential markers for scar presence and location, though these demonstrate less obvious shifts. If the presence of scar is already known, $dT_{\text{QRSend}}$, $dT_{\text{VCGmean}}$, ΔQRS${}_{\text{area}}$ and ΔQRSd show promising utility in accurately determining the location of the scar, if not precisely the dimensions of the scar.

In this work, it has not been possible to identify a specific range or value of $dT_{\text{QRSend}}$ that underlies arrhythmic risk. However, based on the subtle and occasionally opposing variations in several metrics, it is possible through machine learning to achieve a high degree of accuracy for differentiating the simulated scars by location, based on changes in the VCG dipole, measurable through the various metrics. The most common scar locations

### 4.3 Effect of scar on VCG dipole

Scarred tissue represents an absence of myocardial cells, having been replaced with collagenous fibrosis. ECG theory states that the total net electrical dipole sensed by electrodes placed on the torso can be thought of as the summation of the local dipoles associated with the electrical activation wavefront (or repolarisation waveback) propagating across the myocardial tissue [50]. The VCG, which may be measured directly or derived from the ECG leads, effectively measures the components of this net dipole throughout the cardiac cycle [50]. Thus, as specific regions of the ventricles lose myocardial mass, they correspondingly lose dipole signal to contribute to the net dipole. During activation, as the wavefront primarily propagates in the endo-epicardial direction, the net dipole is orientated away from the scarred region, towards tissue with preserved myocardial (and dipole) mass. As we have shown in this work, this movement of the net dipole due to a loss of myocardial dipole signal in regions of scar leads to a noticeable change on the VCG, most evident in the VCG vector loops (Fig. 4).

The unpredictable effects of changes in UVC parameters indicates a truth that is confirmed by the effects of scar density on the dipole action: the action of scar is a complicated and unpredictable process. On the one hand, one has the known conduction slowing effect of scar. This would, all things being equal, result in a slower depolarisation and repolarisation, an effect that is more exaggerated at fast pacing rates. For scar in both the LV free wall and the septum, this would be expected to cause the dipole to ‘linger’. However, there is a competing action: the mass of excitable tissue, which is reduced in the presence of scar, as discussed above. As such, this presents an opposing action, wherein the slowing effects of scar are reduced, due to the reduced excitable mass reducing the effect on dipole action. Consequently, there is a ‘sweet spot’ in the scar mass, wherein the dipole action is at its maximum—the conduction slowing effects of scar are not counter-balanced by the effect of reduced excitable mass. Furthermore, increased fibrosis increases the likelihood of wavefront fractionation, which can potentially cause opposing contributions to the dipole along the non-parallel wavefront, resulting in a reduced dipole action. Due to these competing actions, the point at which the ‘maximum’ effect occurs is not easy to predict, with counter-intuitive results such as lower density scar having a greater effect on metrics of note.

The utility of $dT_{\text{QRSend}}$ is that it, to a large degree, avoids these complications, and in demonstrating opposing effects for LV free wall scar and septal scar, potentially allows not only for scar detection by VCG analysis, but also scar localisation. Under control conditions, there is an angular difference between the dipole at QRS end for fast and slow pacing. When scar is present in the LV, this results in a reduced excitable mass of tissue, coupled with slowed conduction. At slow pacing, the effect of scar on the VCG loop is reduced, and the location of the dipole at the end of the QRS complex is (relatively) insensitive to scar. However, at fast pacing rates, septal scar causes a slowing of the final repolarisation of the heart, causing an increase in $dT_{\text{QRSend}}$. This can be compared with the effect of LV scar, which instead manifests as a reduced excitable mass of cardiac tissue, and thus reduces the effect of pacing, and reduces $dT_{\text{QRSend}}$.

These differences in the VCG dipole upon fast pacing, between different scar locations, were seen to be important and potentially significant. It was decided that determining how these differences could be utilised to their greatest effect could most efficiently be achieved using machine-learning techniques: the opposing actions of scar on VCG dipole can be teased out when several metrics are combined, rather than assessed individually. $dT_{\text{QRSend}}$ as may be expected, is the most relatively valuable metric in random forests, but this is closely followed by ΔWAE and ΔQRS${}_{\text{area}}$. These latter two metrics, especially the former, are far less individually sensitive than $dT_{\text{QRSend}}$, but take average properties of the entire QRS complex, and the subtle changes in these metrics are important as discriminators which result in an accurate tool to determine scar location. Machine learning techniques in general, as shown here with random forests, thus present a potentially useful clinical tool to effectively combine the information from several VCG metrics, and thus a more universal representation of the VCG dipole, to accurately assess the effect of scar on the VCG dipole.

### 4.4 Clinical applications

While promising work in identifying scar location and properties from ECG recordings has been achieved [51], intra-patient differences in ECG and VCG traces and metrics are significant [52], and so using these signals to reliably identify potentially arrhythmogenic scar in sinus rhythm may be problematic. However, looking for *differences* in the same patient’s traces (or metrics derived from them) between beats at different pacing rates, which may identify arrhythmic markers, represents a more clinically feasible scenario; heart rate dependent changes in VCG metrics have previously been noted clinically, though this was observed in healthy hearts [37], [53].

One of the key limitations of recent approaches to identify arrhythmogenic scar substrates in NICM patients is the need for catheterisation, and to perform invasive mapping or programmed stimulation strategies to allow BCLs of up to 200ms (approximately 300 bpm)—much faster than physiological rates. The main benefit of the methodology proposed here is to perform non-invasive VCG analysis; however, it also requires both slow and fast sinus rhythm pacing. Increases in sinus heart rate, up to approximately 180bpm, may be achieved clinically using exercise testing, which is a commonly-used provocative stress test performed to assess different aspects of cardiac function [54], [55], [56]. Alternatively, heart rate may be elevated to similar levels using pharmacological means.

Although in this work the model was paced relatively rapidly (300ms BCL, equivalent to 200 bpm), it is probable that potential NICM device candidates may show more marked rate-dependent conduction slowing than represented here, as has been suggested in ex-vivo experimental optical mapping studies [57]. Our aim in this study was to separately probe the direct effects of scar-related conduction slowing on the VCG, and thus the myocardial cells in our model were represented by healthy ionic properties. However, it is thought that fully representing possible ionic remodelling seen in heart failure within the model may allow a more marked conduction slowing through the scar to be brought about at relatively slower pacing rates of approximately 500ms BCL (120 bpm). This suggests the feasibility of this approach to be used clinically in these patients using exercise testing or pharmacological provocation. However, due to lack of comprehensive data on such remodelling in this population, we were unable to represent these effects at this stage.

## 5 Limitations

As a proof-of-concept study, we have used a single (non electrically personalised) cardiac model, with idealised geometries and representations of scar. While permitting a simplified analysis and facilitating interpretation of simulation results, it does limit the direct clinical applicability of the results. Future work would thus look to remove the idealisations used in this study to enhance the clinical utility, no longer constrained by the generality required of a proof-of-concept study. Perhaps the most pressing adjustment for increased clinical relevance would be to investigate more realistic scar geometries. This could be achieved through further investigation of simulated scar geometries, expanding to include multiple scar locations, or using scar geometries derived from clinical observations; further consideration of scar heterogeneities would be invaluable. A further limitation of the current work is the use of a single torso model—future work could investigate the influence of a more varied patient cohort, ensuring the generality of the results to a wider cross-section of potential patients. With suitable data, the use of clinical ECG recordings would ensure clinical relevance.

A further simplification to increase the computational tractability of this study was that we used the known significant rate-dependent conduction slowing shown to be associated with arrhythmia risk in our previous computational modelling study [4] and other clinical studies [3], [5] rather than directly performing computationally-intensive arrhythmia induction protocols on the model. Nonetheless, we have used our analysis to highlight the potential importance of $dT_{\text{QRSend}}$ as an means of (non-invasively) quantifying subtle but important changes in conduction slowing through scarred tissue in NICM, which may not be visible with other measurement techniques; the potential visibility is enhanced when several metrics are combined in random forests. We therefore see the utility of this work in generating an hypothesis that might be clinically tested in the future on a NICM patient cohort.

## 6 Conclusions

Differences in the electrophysiology between fast and slow pacing can be illuminated by quantifiable, measurable VCG metrics. With some of these metrics being more affected than others by the patho-electrophysiological properties of scar, this opens up an avenue for non-invasive detection, and potentially even localisation, of scar in the heart using readily available clinical apparatus. This work indicates that changes in the angular difference between the electrical dipole of the heart at the end of the QRS complex represent a promising candidate for such a clinically relevant metric, with supporting information given by the angular difference between the mean dipoles and the maximum dipoles, as well as changes in the maximum dipole magnitude, the QRS duration and the area under the VCG loop. Furthermore, machine learning in the form of random forest analysis presents a potentially useful tool to accurately and efficiently determine the properties of a scar to a greater degree of granularity than is possible using single VCG metrics.

## Declaration of Competing Interest

The authors declare that they have no known competing financial interests or personal relationships that could have appeared to influence the work reported in this paper.

## Appendix A Supplementary data

MMC S1
