# Supplementary material for: A computational investigation into rate-dependant vectorcardiogram changes due to specific fibrosis patterns in non-ischæmic dilated cardiomyopathy
Source: Comput Biol Med. 2020 Aug;123:103895. doi: 10.1016/j.compbiomed.2020.103895 (PMC7429989; doi:10.1016/j.compbiomed.2020.103895)
Supplement: MMC S1 [file mmc1.pdf]

A computational investigation into rate-dependant vectorcardiogram changes due to specific fibrosis patterns in non-ischæmic dilated cardiomyopathy: Supplementary Information

Philip M. Gemmell[1,*], Karli Gillette[2], Gabriel Balaban[3], Ronak Rajani[1], Edward J. Vigmond[4], Gernot Plank[2], and Martin J. Bishop[1]

[1]*King's College London*
[2]*Medical University of Graz*
[3]*University of Oslo*
[4]*University of Bordeaux*
[*]*Corresponding author: philip.gemmell@kcl.ac.uk*

11th June 2020

A Effect of scar size and morphology on rate-dependent changes in VCG metrics

Those metrics that were determined to be of less use in predicting scar properties are: $\Delta\overline{\mathrm{VCG}}$ ΔWAA, ΔWAE, $\Delta t_{\mathrm{VCGmax}}$ dT_{QRSstart} and dT_{QRSend}. The effect of the presence of the largest simulated scar (for both LV free wall and septum) on the differences for these metrics is shown in Fig. A.1. All of these metrics, save for $\Delta t_{\mathrm{VCGmax}}$ show relatively little change compared between the presence or absence of scar in this cases; the large change in $\Delta t_{\mathrm{VCGmax}}$ is deceptive in the same manner as the change noted in $\Delta\mathrm{VCG}_{\mathrm{max}}$ in the main text, and for the same reasons.

As in the main text for the significant metrics, more in depth analysis of the metrics is conducted to investigate the individual UVC parameter effects: the effect of scar volume and scar surface area is shown in Fig. A.2, whereas the effect of the various scar size parameters (ϕ, ρ, z) is shown in Fig. A.3; in the latter, all parameters save the one being considered are set to their maximum value. The effects on each metric shall now be discussed in turn.

A.1 Effects on $\overline{\mathrm{VCG}}$

$\Delta\overline{\mathrm{VCG}}$ shows little correlation with scar size, as measured by volume or area, or morphology, for any of the UVC parameters, for scar in the LV.

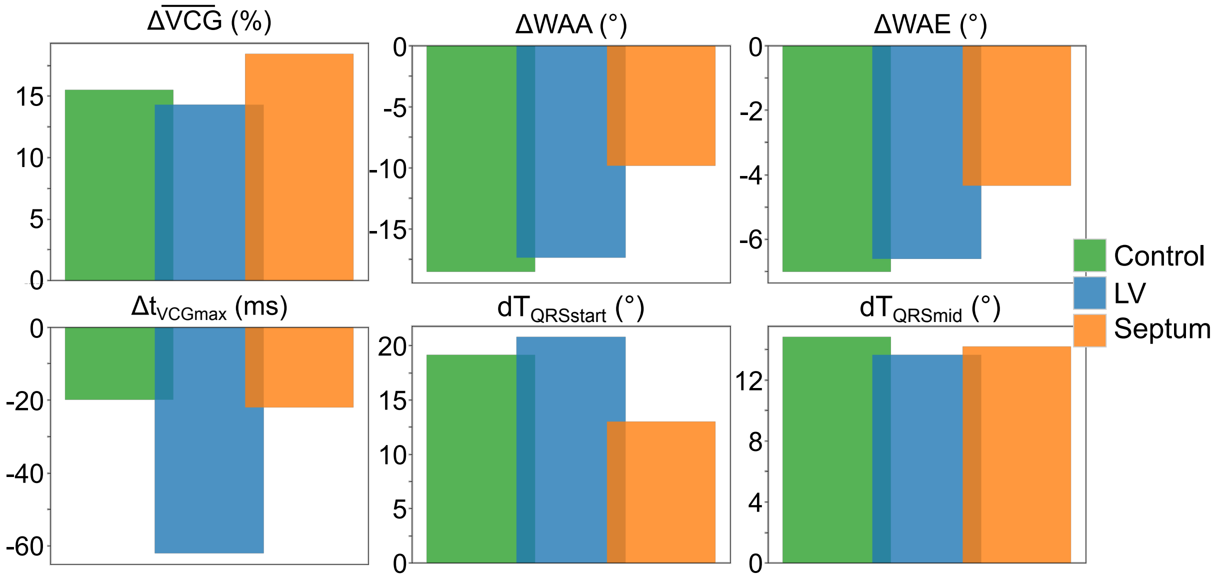

Figure A.1: Changes in metrics between fast and slow pacing. Changes in the $\Delta\overline{\text{VCG}}$ are expressed as percentage changes from their value for BCL=600 ms.

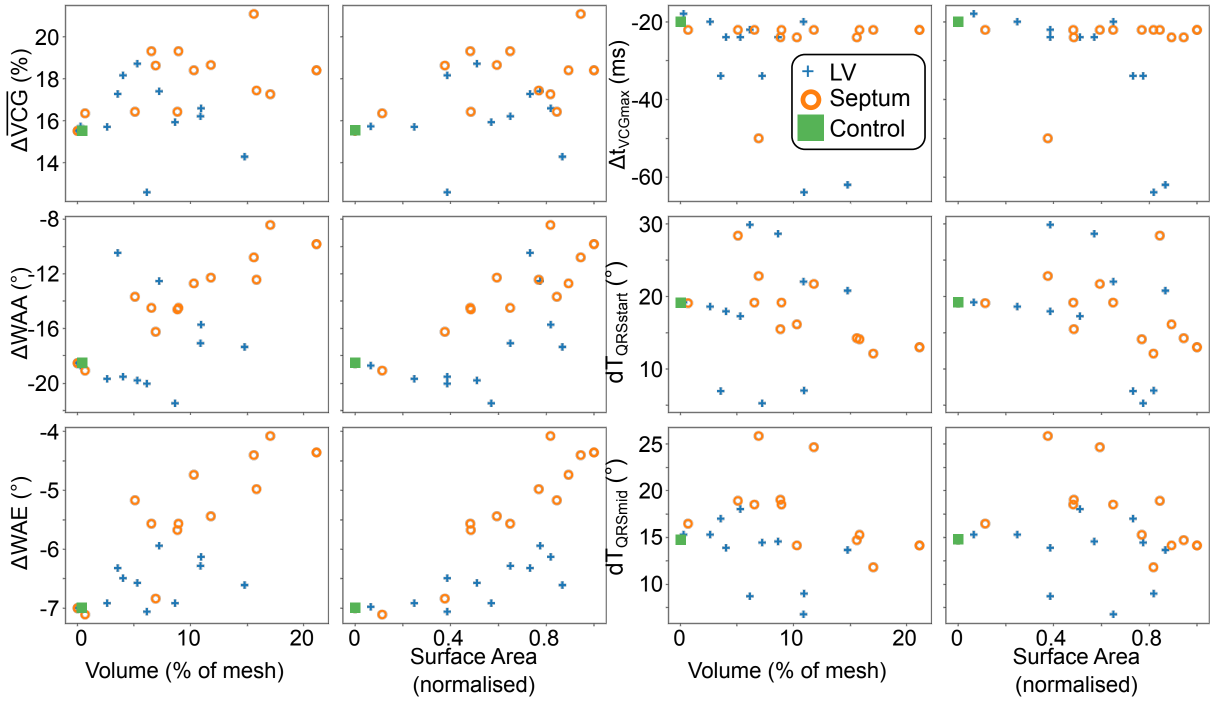

Figure A.2: Changes in metrics between fast and slow pacing versus volume and surface area of scar, for both septal and LV free wall scars. $\Delta\overline{\text{VCG}}$ is expressed as percentage changes from slow pacing, Δt_{VCGmax} is expressed as the difference between slow and fast pacing, and ΔWAA, ΔWAE, dT_{QRSstart} and dT_{QRSmid} are expressed as angular differences between the dipole vectors.

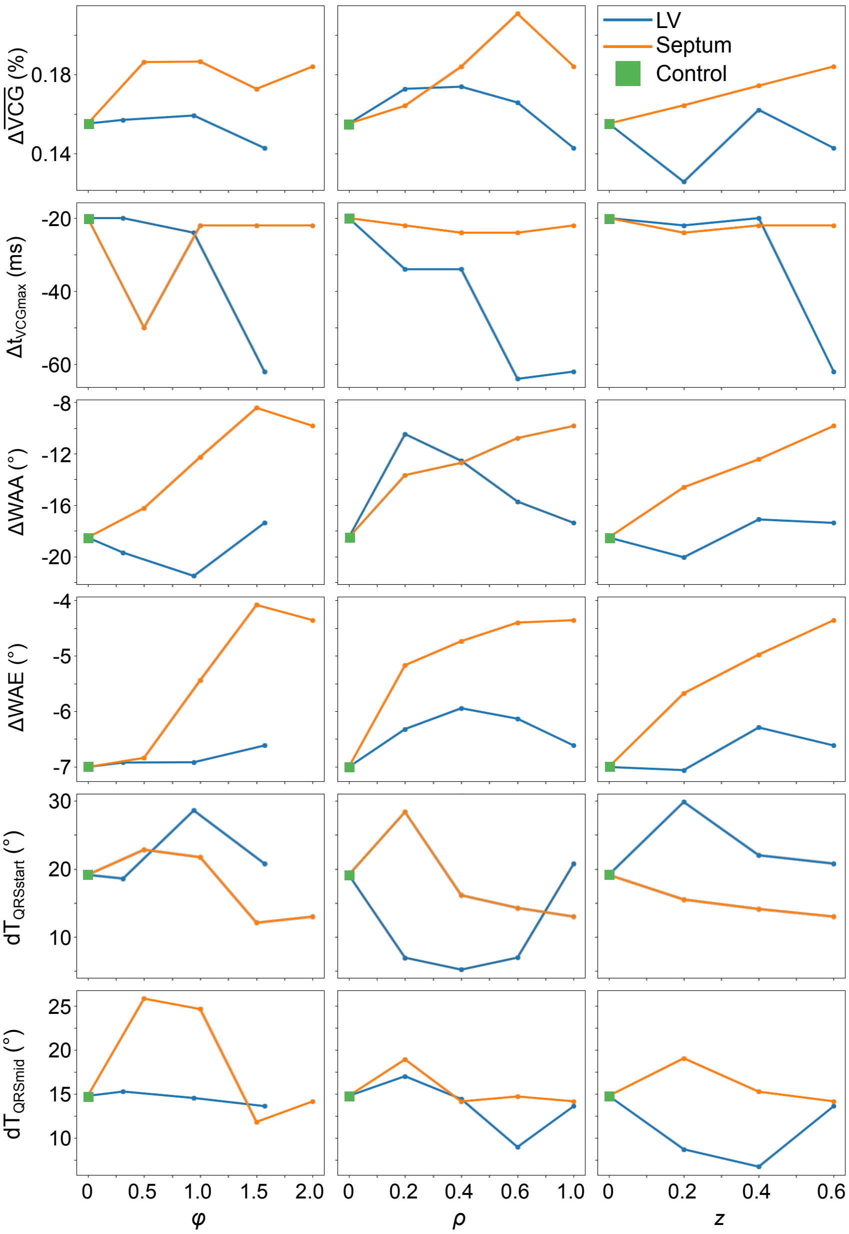

Figure A.3: Changes in metrics between fast and slow pacing versus ϕ, ρ and z, for both septal and LV free wall scars. $\Delta\overline{\mathrm{VCG}}$ is expressed as percentage changes from slow pacing, $\Delta t_{\mathrm{VCGmax}}$ is expressed as the difference between slow and fast pacing, and ΔWAA, ΔWAE, dT_{QRSstart} and dT_{QRSmid} are expressed as angular differences between the dipole vectors.

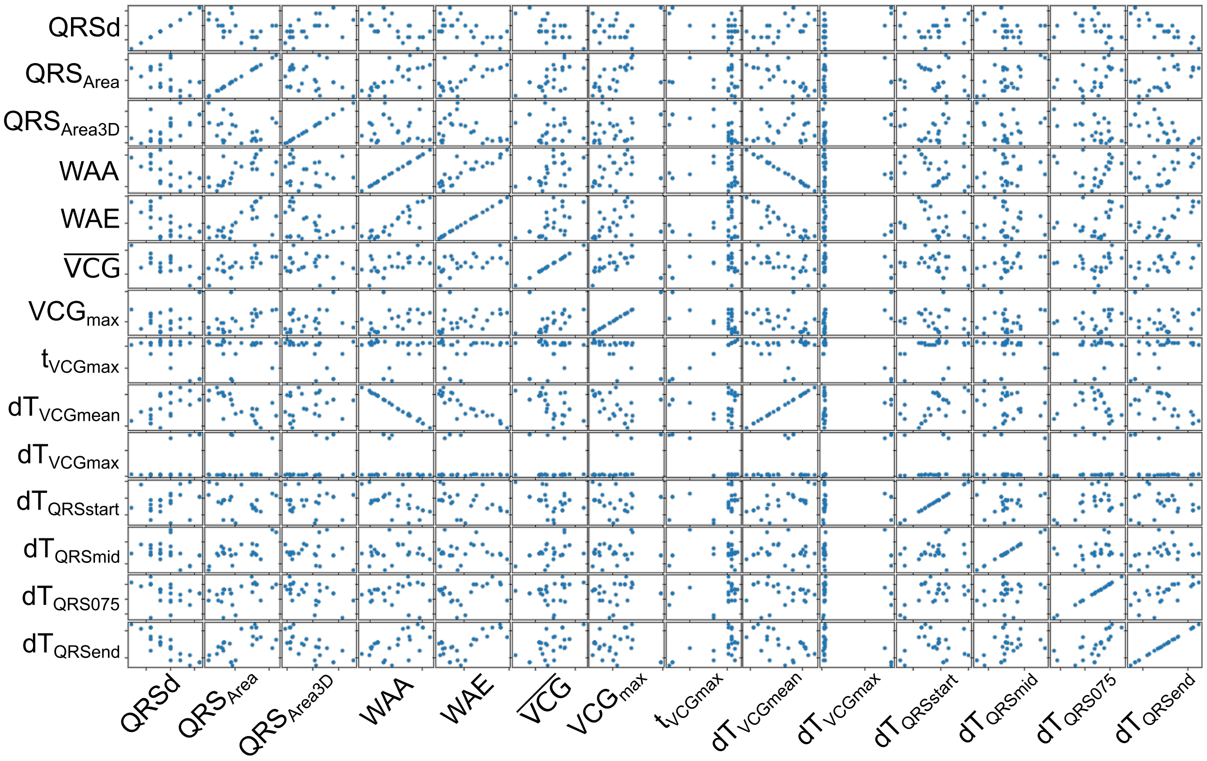

Figure A.4: Correlation between measured metrics.

There does, however, seem to be a positive correlation when the scar is in the septum. However, this correlation is relatively weak in terms of both volume and area. When assessed by the UVC parameters, there is only a consistent positive correlation for z; for both ϕ and ρ, an increase in the scar size does not consistently lead to an increase in $\Delta\overline{\mathrm{VCG}}$. Furthermore, the percentage change is marginal at best—the control change is 15.5%, while the maximum observed is 19.5%.

A.2 Effects on t_{VCGmax}

There is negligible change under most conditions in $\Delta t_{\mathrm{VCGmax}}$, save for some isolated points at which there is a substantial change; these substantial changes are not necessarily correlated with scar size, displaying only some level of correlation for LV scar. However, as discussed in the main text, this is due to a shift in the overall loop dynamics of the VCG, and thus a shift at which stage in the VCG loop $\mathrm{VCG}_{\mathrm{max}}$ occurs. Without substantially more work, it is impossible to say with certainty whether this dynamic shift can be used as an accurate predicator of scar presence.

A.3 Effects on Weighted Angular Elevation and Azimuth

The WAA and WAE metrics are referred to such to avoid confusion with the rotational parameter for scar size ϕ.

The change in WAE shows a relatively consistent increase with scar size, which is both more consistent and more noticeable for scar in the septum than for scar in the LV free

wall. However, the change is marginal, regardless, from $-7°$ for no scar, to a maximum of $-4°$; such a marginal difference of only $3°$ between control and maximum observed effect is unlikely to translate to a useful clinical indicator.

There is relatively little correlation between scar size and either increase or decrease in WAA for scar in the LV free wall, for either scar size (assessed by either volume or area) or morphology (ϕ, ρ or z). There appears to be a far more promising correlation for scar in the septum, however, with a consistent positive correlation (save for a marginal decline at maximum ranges of ϕ). Furthermore, the differences involved are greater than those for WAE, thus representing a more promising clinical target ($-18°$ for control, $-8°$ maximum change). However, it was determined that WAA and dT_{VCGmean} demonstrated almost perfect negative correlation (Fig. A.4), and dT_{VCGmean} was chosen as the candidate for further investigation.

A.4 Effects on dT_{QRSstart} & dT_{QRSmid}

There is negligible correlation between scar size and either dT_{QRSstart} or dT_{QRSmid}. This is whether the scar size is judged by either volume, area, or one of the UVC parameters.
